# Anterior Mandibular Osteomyelitis: A Narrative Review of Clinical Presentation, Diagnosis, and Management Strategies

**DOI:** 10.7759/cureus.86667

**Published:** 2025-06-24

**Authors:** Munish Kumar, Mohit Verma, Neeraj C Attri, Fayaz Alam, Harleen Kaur, Rishabh Kasrija, Seema Gupta

**Affiliations:** 1 Department of Oral and Maxillofacial Surgery, Guru Nanak Dev Dental College and Research Institute, Sunam, IND; 2 Department of Oral and Maxillofacial Surgery, Jagadguru Sri Shivarathreeshwara Dental College, Mysuru, IND; 3 Department of Orthodontics, Kothiwal Dental College and Research Centre, Moradabad, IND

**Keywords:** anterior, management, mandible, osteomyelitis, review

## Abstract

Osteomyelitis of the anterior mandible is a relatively uncommon but clinically significant condition that presents unique diagnostic and therapeutic challenges. Although mandibular osteomyelitis more frequently affects the posterior region, anterior involvement is increasingly recognized owing to rising incidences associated with dental implants, trauma, and systemic conditions such as diabetes and immunosuppression. This narrative review provides a comprehensive overview of anterior mandibular osteomyelitis, highlighting its pathogenesis, epidemiological trends, clinical features, diagnostic approaches, and current treatment modalities. The anatomical and functional significance of the anterior mandible necessitates early recognition and precise management to prevent long-term morbidities. The clinical manifestations vary from acute infections with swelling and pain to chronic presentations involving bone necrosis, sinus tract formation, or features mimicking neoplastic processes. Advanced imaging techniques, including cone-beam computed tomography and magnetic resonance imaging, are crucial for the early detection and assessment of disease extent, particularly in chronic or atypical cases. Management typically involves a multidisciplinary strategy that combines prolonged antibiotic therapy with surgical debridement or resection, particularly in chronic or refractory cases. Emerging techniques such as distraction osteogenesis and computer-assisted surgical planning have improved outcomes in cases requiring reconstruction. Conservative approaches may be considered for early or non-suppurative forms of the disease, while hyperbaric oxygen therapy has shown promise in select patients. This review highlights the importance of clinician awareness regarding the various presentations of anterior mandibular osteomyelitis. Emphasis should be placed on comprehensive evaluation, prompt intervention, and addressing predisposing factors to reduce recurrence and enhance prognosis. Further studies are warranted to better define the anterior-specific epidemiological data, microbial profiles, and long-term outcomes, ultimately supporting the development of targeted management protocols.

## Introduction and background

Osteomyelitis is a debilitating inflammatory condition of the bone and marrow, predominantly caused by bacterial infection, leading to progressive bone destruction and necrosis if left untreated [1]. While osteomyelitis can affect any bone, mandibular involvement is particularly concerning due to the region's high vascularity, functional significance, and proximity to critical anatomical structures [2]. The anterior mandible, though less frequently affected than the posterior region, presents distinct clinical challenges due to its aesthetic importance, dense cortical bone structure, and limited accessibility for surgical intervention [2,3]. The limited accessibility of the anterior mandible for surgical intervention is primarily due to its anatomical proximity to vital structures such as the mental nerve, the thin overlying mucosa, and its location within the aesthetic zone, where surgical exposure and manipulation must be performed with care to avoid nerve injury, scarring, or disfigurement. Additionally, the dense cortical bone and restricted soft tissue coverage in this region can make flap elevation and debridement more technically challenging compared to the posterior mandible [2].

Anterior mandibular osteomyelitis poses greater challenges than posterior cases due to its location in the facial aesthetic zone, proximity to the mental nerve, and dense cortical bone, which complicate both medical and surgical management. Limited soft tissue access and the need to preserve functions like speech, lip support, and appearance make surgical intervention more delicate. These factors require a precise, multidisciplinary approach for effective treatment and reconstruction. The condition may present in acute, subacute, or chronic forms, with clinical manifestations varying from localized pain, swelling, and pus discharge to severe complications such as pathological fractures, fistulae, and systemic infection [4]. Given its potential for significant morbidity, early recognition and targeted management are essential to prevent irreversible bone loss and functional impairment.

The etiology of anterior mandibular osteomyelitis is multifactorial, with odontogenic infections being the most common predisposing factor, often arising from untreated dental caries, periodontal disease, or periapical abscesses [5]. Trauma, including fractures and surgical interventions such as dental implant placement or extractions, can also introduce pathogens into the bone, triggering infection [6]. Systemic conditions such as diabetes mellitus, immunosuppression, and malnutrition further exacerbate susceptibility by impairing host immune responses and bone healing [7]. Additionally, compromised blood supply, whether due to radiation therapy, vascular disease, or anatomical variations, can predispose the anterior mandible to ischemic necrosis and subsequent infection [8]. Anatomical variations that pose a higher risk include a dominant reliance on a single nutrient artery, such as the inferior alveolar artery, hypoplastic or absent collateral vessels like the submental and facial arteries, and variations in the mandibular foramen or canal anatomy that may limit effective vascular perfusion. These factors can reduce the region's ability to recover from trauma or infection, increasing the risk of osteomyelitis. The interplay of these factors underscores the need for a thorough patient history and comprehensive diagnostic evaluation to identify underlying risk factors and guide appropriate treatment.

Diagnosing osteomyelitis of the anterior mandible requires a combination of clinical, radiographic, and microbiological assessments. Conventional radiographs, though initially useful, often fail to detect early osteolytic changes, necessitating advanced imaging modalities such as cone-beam computed tomography (CBCT), magnetic resonance imaging (MRI), or scintigraphy for accurate evaluation of bone involvement [9]. Histopathological examination and microbial cultures are critical for confirming the diagnosis and identifying causative pathogens, particularly in chronic or refractory cases where atypical organisms or biofilm formation may complicate treatment [10].

The most common clinical signs and symptoms of anterior mandibular osteomyelitis include localized pain, swelling, tooth mobility, purulent discharge, and non-healing extraction sites. In more advanced or chronic cases, patients may present with fistula formation, facial asymmetry, or pathological fractures. Acute anterior mandibular osteomyelitis typically presents with the rapid onset of pain, swelling, and possible systemic symptoms, such as fever, indicating an active infection. In contrast, chronic cases tend to have a more indolent course with signs such as bone necrosis, sinus tract formation, and features that may mimic tumors or granulomatous diseases. Early recognition of acute symptoms is essential to prevent progression to the chronic stage [2].

Management strategies for anterior mandibular osteomyelitis are multidisciplinary, incorporating antimicrobial therapy, surgical debridement, and adjunctive treatments such as hyperbaric oxygen therapy (HBOT) or reconstructive surgery in advanced cases [11]. Conservative approaches, including the use of antibiotics and anti-inflammatory agents, may be effective in early or non-suppurative forms of anterior mandibular osteomyelitis, helping to control infection and prevent progression without the need for surgery. In select, particularly refractory cases, HBOT has shown promising outcomes [12]. Surgical intervention, ranging from sequestrectomy to segmental resection with reconstruction, may be required in cases of extensive bone loss or persistent infection [13]. Reconstructive approaches must balance infection control with optimal rehabilitation because premature or aggressive reconstruction in the presence of unresolved infection can lead to graft failure, persistent inflammation, or recurrence of osteomyelitis. At the same time, delaying reconstruction for too long can result in significant bone and soft tissue loss, compromising facial aesthetics, speech, mastication, and overall quality of life. In the anterior mandible, where both cosmetic appearance and oral function are critical, achieving this balance is essential. Effective infection control ensures a stable and healthy tissue environment, while timely and well-planned reconstruction, utilizing techniques such as vascularized bone grafts or distraction osteogenesis, restores structural integrity and function without increasing the risk of complications.

This narrative review aimed to consolidate current knowledge on anterior mandibular osteomyelitis, focusing on its clinical presentation, diagnostic challenges, and evolving management strategies. By synthesizing evidence from contemporary literature, this review aimed to enhance clinician awareness of both typical and atypical presentations, ultimately improving diagnostic accuracy and therapeutic outcomes for affected patients.

## Review

Methodology

This narrative review employed a systematic approach to literature retrieval, ensuring a comprehensive synthesis of the available evidence on anterior mandibular osteomyelitis. A literature search was conducted across multiple electronic databases, including PubMed, Scopus, Web of Science, and Google Scholar, covering publications published from January 1990 to October 2024. The search strategy was designed to identify studies relevant to osteomyelitis of the mandible, with a focus on anterior involvement. Medical Subject Headings (MeSH) terms used included "Osteomyelitis", "Mandible", "Anterior Mandible", "Jaw Diseases", "Bacterial Infections", "Dental Implants", and "Immunocompromised Host". Additional keywords, such as "mandibular osteomyelitis", "anterior jaw infection", "actinomycosis", "sclerosing osteomyelitis", AND, OR "pycnodysostosis", were combined using Boolean operators (AND, OR) to refine the search. The inclusion criteria were peer-reviewed articles, case reports, case series, retrospective studies, and systematic reviews published in English, focusing on epidemiology, pathogenesis, diagnosis, and management of mandibular osteomyelitis, particularly in the anterior region. Exclusion criteria included non-English articles, studies focusing exclusively on maxillary or non-mandibular sites, and publications lacking clinical or scientific relevance. A manual review of the reference lists from key articles was conducted to identify additional relevant studies. Data were synthesized narratively to address the multifaceted nature of the condition, with an emphasis on integrating findings from diverse clinical contexts.

Epidemiology

Osteomyelitis of the jaw, which is less prevalent in the antibiotic era, remains a significant clinical concern, particularly in developing regions and immunocompromised populations [2]. The mandible is more frequently affected than the maxilla because of its relatively poor vascular supply, with the posterior region being the most common site [4]. Although less reported, anterior mandibular osteomyelitis is clinically significant and potentially underdiagnosed. Retrospective studies have indicated variable prevalence across demographic groups, with risk factors including dental infections, trauma, and systemic disease. For instance, Metwally et al. [4] reported a notable incidence of jaw osteomyelitis over a five-year period at Cairo University, although anterior-specific data were not isolated. The incidence rate of this condition was observed to be significantly higher in males than in females, particularly within the age bracket of 30-39 years [4]. This ailment is more frequently observed in regions characterized by low socioeconomic status, classifying it as a significant health issue in developing nations [4]. Similarly, Koorbusch et al. [5] and Adekeye and Cornah [6] highlighted odontogenic infections and trauma as key predisposing factors for mandibular osteomyelitis, with anterior involvement noted in specific contexts. Immunocompromised patients, including those with diabetes, chemotherapy, or genetic disorders such as pycnodysostosis, are at a higher risk of anterior mandibular involvement [7]. Sood et al. [7] observed atypical patterns, including anterior jaw infections, in immunocompromised patients. Additionally, the increasing use of dental implants has introduced new risks, as documented in case reports by Balanger et al. [8] and systematic reviews by Kellesarian et al. [9], which have identified osteomyelitis following implant placement in the anterior mandible. Pediatric cases, syndromic conditions, and trauma further contribute to the epidemiology of anterior mandibular osteomyelitis, underscoring the need for targeted studies to quantify its prevalence and clinical course [7,9].

Pathogenesis

The pathogenesis of anterior mandibular osteomyelitis involves a complex interplay between microbial invasion, host immune response, and local anatomical factors. The primary etiological pathway is the contiguous spread of infection from odontogenic sources, such as periapical abscesses, periodontal infections, or complications, following dental extractions [2]. These infections progress through the medullary cavity, leading to vascular compromise, thrombosis, ischemia, and bone necrosis. Although the anterior mandible is relatively well vascularized compared to the posterior region, systemic conditions such as diabetes, immunosuppression, or metabolic bone disorders can exacerbate susceptibility [5,7]. Microbial invasion by both aerobic and anaerobic bacteria, including *Streptococcus, Staphylococcus, *and* Actinomyces*, initiates the disease process [12]. Actinomycotic infections, noted for their chronic and indolent course, are particularly significant in the anterior mandible and often mimic malignancy or granulomatous disease [12]. Trauma, including anterior mandibular fractures or surgical interventions such as implant placement, also contributes to pathogenesis by introducing infection or compromising bone integrity [9]. Systemic conditions and syndromic disorders, such as pycnodysostosis, alter bone architecture and increase infection risk even without overt trauma [13]. Vascular compromise, despite collateral blood flow from the submental and facial arteries, creates a hypoxic microenvironment that is conducive to bacterial growth and sequestrum formation. Histologically, the condition is characterized by bone destruction, inflammatory infiltrates, microbial colonies, and necrotic bone, with chronic forms exhibiting fibrosis and the formation of sinus tracts [2,5].

Diagnostic approaches

**Figure 1:**
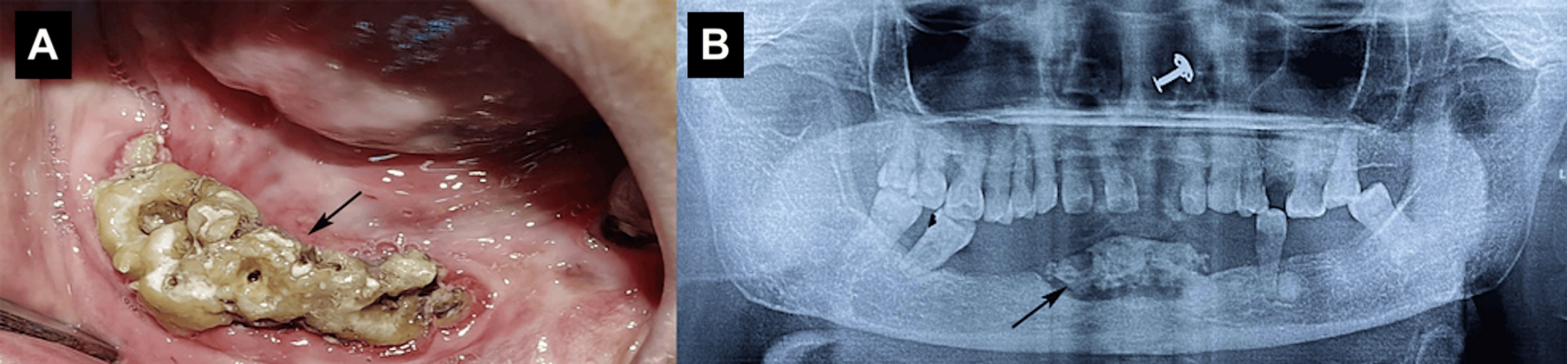
Chronic osteomyelitis of the anterior mandible (A), necrotic bone exposed intraorally; (B) dental panoramic tomograph (DPT) showing sequestered bone. This figure represents the author's own clinical case, and it is published with the patient's written informed consent.

Accurate diagnosis of anterior mandibular osteomyelitis is critical for preventing its progression to chronic infection or structural compromise [2]. The diagnostic process integrates clinical evaluation, imaging, microbiological analysis, and histopathological examination [1]. Clinically, patients present with pain, swelling, tooth mobility, purulent discharge, or non-healing extraction sites [10]. Chronic cases may exhibit fistula formation, pathological fractures, or facial asymmetry [2]. A thorough history, including recent dental procedures, trauma, and systemic diseases, is essential [5]. Radiographic imaging plays a pivotal role with traditional radiographs, such as periapical and panoramic views, which are often inadequate for early detection [5]. Computed tomography (CT) and CBCT provide high-resolution imaging of cortical destruction and sequestrum formation, whereas MRI is used to detect early marrow edema and distinguish infection from malignancy [11]. Kaneda et al. [11] demonstrated MRI's superiority in mandibular osteomyelitis for early detection of mandibular osteomyelitis. Microbiological analysis of aspirates or biopsy specimens has identified causative organisms, with *Staphylococcus aureus*,* Streptococcus species*,and* Actinomyces* commonly isolated [12]. Prolonged anaerobic culture is necessary for actinomycotic infections [12]. Histopathological examination confirmed necrotic bone, inflammatory infiltrates, and microbial colonies, particularly in atypical presentations, such as sclerosing osteomyelitis. Laboratory markers such as erythrocyte sedimentation rate (ESR), C-reactive protein (CRP), and white blood cell (WBC) count support diagnosis but are nonspecific, serving primarily to monitor treatment response. Combining multiple diagnostic modalities ensures a comprehensive assessment of the disease extent and etiology (Figure 1) [13].

Management and treatment

Management of anterior mandibular osteomyelitis requires a tailored approach that combines antimicrobial therapy, surgical intervention, and correction of predisposing factors [2]. Empirical broad-spectrum antibiotics, such as penicillin, clindamycin, and metronidazole, are promptly initiated, followed by culture-directed therapy [2]. Chronic cases often require prolonged courses (four to six weeks), with actinomycotic infections necessitating high-dose penicillin for weeks to months [12]. A previous study advocated for the continuation of postoperative treatment for a duration of two to four months following the alleviation of symptoms [14]. It has been posited that the synergistic application of antibiotic therapy alongside surgical intervention yields significant efficacy in the management of chronic suppurative osteomyelitis [14].

Surgical debridement is critical in chronic or suppurative cases to remove necrotic bone and drain purulent collections [3]. The procedures range from sequestrectomy and curettage to segmental resection with reconstruction using bone grafts or vascularized flaps [15]. Anterior mandibular involvement requires careful surgical planning to preserve facial aesthetics, mental nerve function, and speech [15]. Xie et al. [16] performed distraction osteogenesis reconstruction of bone defects created after surgical resection of the left side of the mandible due to chronic osteomyelitis. It has been documented that the implementation of trifocal distraction osteogenesis in the context of mandibular body defects, resulting from the excision of ameloblastoma or keratocystic odontogenic tumors, can facilitate satisfactory new bone formation and subsequent dental rehabilitation [17]. Furthermore, through the lengthening of the mandible by 6.5 cm, distraction osteogenesis has effectively addressed mandibular body defects that are secondary to oncologic interventions in the head and neck region [18].

Advances in computer-assisted visual surgical planning (VSP) and three-dimensional (3D) printing have improved reconstructive outcomes [19]. The advantages derived from the utilization of VSP/3D-printed guides/plate reconstruction include enhanced efficiency, reproducibility, and augmented precision. Nonetheless, these benefits require careful evaluation in light of their associated financial implications. The additional commercial expenditure associated with VSP/3D-printed guides/plates has been documented to be approximately $4,000 [20], with potential cost reductions achievable if segments of the process are conducted internally. Efficiency improvements in surgical duration have been reported to vary between 80 and 88 minutes [21], while enhancements in ischemic time have been observed to range from 36 to 50 minutes [22]. The reduction in operating room costs has been demonstrated to counterbalance the supplementary expenses related to both the VSP and 3D printing methodologies [23].

Conservative management with antibiotics and anti-inflammatory medications may suffice in early or non-suppurative cases, such as primary chronic or sclerosing osteomyelitis [2]. Studies evaluating HBOT have reported benefits such as reduced infection recurrence, enhanced wound healing, improved bone regeneration and revascularization, and shorter duration of systemic antibiotic therapy. Additionally, patients often experience resolution of clinical symptoms, such as pain, swelling, and sinus tract discharge. These outcomes support the use of HBOT as a valuable adjunctive treatment in complex or immunocompromised cases where conventional therapy alone may be insufficient [24]. A multifaceted approach involving rigorous surgical irrigation and meticulous bone debridement, along with the administration of intravenous antibiotic therapy and prompt initiation of HBOT, is essential for compromised patients who present with recurrent episodes of osteomyelitis [24]. Addressing underlying risk factors, such as poor oral hygiene, dental infections, and systemic diseases, is crucial for preventing recurrence [3]. Prosthesis removal is often necessary [9]. Long-term follow-up with clinical examination, imaging, and laboratory monitoring ensured disease resolution and functional restoration.

Management strategies for anterior mandibular osteomyelitis are generally applicable globally but often depend on regional healthcare resources. Advanced interventions like imaging, surgical reconstruction, or HBOT may be limited in low-resource settings, necessitating modified treatment plans. Poor dental health, including periodontal disease and untreated caries, significantly increases the risk of osteomyelitis by serving as a source of chronic infection. These conditions facilitate bacterial invasion into the bone, especially following extractions or in immunocompromised individuals, making dental health a key factor in both prevention and disease progression [5].

Discussion

Osteomyelitis of the anterior mandible, though less common than posterior mandibular involvement, presents unique challenges owing to its anatomical, functional, and aesthetic significance [1]. The mandible's limited vascularity predisposes it to infection, but the anterior region's collateral blood supply offers some protection, which can be compromised by systemic risk factors, such as diabetes or immunosuppression [7]. The increasing prevalence of dental implants has introduced new risks, with peri-implant infections emerging as a significant cause of anterior mandibular osteomyelitis [9].

Clinical presentations vary widely from acute suppurative infections to chronic actinomycotic or sclerosing variants that mimic neoplasms or granulomatous diseases [2]. Key differential diagnoses to consider when evaluating suspected anterior mandibular osteomyelitis include neoplastic conditions (e.g., osteosarcoma, metastatic lesions), granulomatous diseases (such as tuberculosis or actinomycosis), sclerosing osteomyelitis, and odontogenic infections. Distinguishing between these conditions is essential and often requires advanced imaging, microbiological testing, and histopathological analysis. Diagnostic challenges are compounded by the limitations of conventional radiography, necessitating the use of advanced imaging techniques, such as MRI and CT, for early detection [11]. Therapeutically, a combination of prolonged antibiotics and surgical intervention remains the cornerstone, with conservative approaches being viable in select cases [5]. Reconstructive challenges in the anterior mandible require multidisciplinary inputs to balance functional and aesthetic outcomes [15].

The gaps in the literature, particularly regarding anterior-specific epidemiology and long-term outcomes, highlight the need for targeted research. Addressing predisposing factors is critical for reducing recurrence rates and optimizing patient care [25]. The recurrence rate of mandibular osteomyelitis was 50% when treated with marginal resection, while it was reduced to 5.5% by segmental osteotomy [2,15]. Non-vascularized osseous grafts possess the capacity to promptly rehabilitate mandibular defects exceeding 6 cm, attributable to benign pathological entities; nonetheless, the probability of failure increases with the utilization of larger grafts [26]. The efficacy of vascularized bone grafting is notably high, establishing it as the preferred therapeutic approach in instances requiring primary reconstruction, particularly for patients who have undergone prior radiation therapy or when concurrent soft tissue replacement is essential. Furthermore, vascularized bone grafts are deemed the optimal solution for mandibular reconstructions that exceed 9 cm in length [27].

## Conclusions

Although relatively rare, anterior mandibular osteomyelitis poses significant diagnostic and therapeutic challenges owing to its diverse clinical manifestations and anatomical complexity. Prompt recognition, supported by advanced imaging, microbiological analysis, and histopathological confirmation, is essential for preventing chronicity and functional impairment. A multidisciplinary approach integrating antimicrobial therapy, surgical intervention, and management of the underlying conditions is key to achieving favorable outcomes. Further research is needed to elucidate anterior-specific trends in the incidence, microbial patterns, and treatment efficacy, which will inform the development of region-specific management protocols. Increased clinical awareness and reporting will enhance understanding and management of this debilitating condition.
